# Biomechanical Performance of Mandibular Molars with Deep Mesio-Occlusal-Distal Cavities Rehabilitated with Horizontal Posts: A 3D Finite Element Analysis

**DOI:** 10.1155/2023/3379373

**Published:** 2023-04-15

**Authors:** Sonali Sharma, Sindhu Ramesh, Jasmine Rayapudi

**Affiliations:** Conservative Dentistry and Endodontics, Saveetha Dental College and Hospitals, Saveetha Institute of Medical and Technical Sciences (SIMATS), Saveetha University, Chennai, India

## Abstract

**Aim:**

To compare and contrast by three-dimensional finite element analysis the biomechanical performance of deep mesio-occlusal-distal cavities of mandibular molars reinforced by different sizes of horizontal fiber posts.

**Materials and Methods:**

The finite element (FE) stress analysis was performed with the ANSYS, a commercial finite element method package. Based on the evidence-based scientific data and on the mechanical properties of materials, i.e., Young's modulus and Poisson ratio, the model of a mandible and mandibular first molar was replicated. The mandibular molar models replicating the clinical scenarios were simulated, designed, and built, assuming all materials to be homogenous, isotropic, and linearly elastic as follows: Model 1 control: the model of an intact first mandibular molar. Model 2: the prepared cavity mesio-occlusal-distal is replicated by the subtraction Boolean method. The remaining thickness of dentin is 1 mm. Model 3: these were rehabilitated by three different diameters of two horizontal fiber posts. Model 3A: fiber post diameter 1 mm, Model 3B: 1.5 mm and Model 3C: 2 mm. The dimensions of the cavity, the intercuspal distance between buccal walls and lingual walls, and the distance of placement of the post from occlusal reference points were all kept constant for all three subgroups of Model 3. The cavities of Model 3 were restored with Filtek bulk-fill posterior composite. After meshing the models, loads were defined on the buccal and lingual distal cusps with a constant value of 600 N and at an angle of 45°.

**Results:**

The results of finite element analysis are expressed as stresses, i.e., tensile compressive, shear, or a combination known as von Mises stresses. The overall von Mises stresses were as follows: Model 1:154.83 Mpa; Model 2: 376.877 Mpa; Model 3A: 160.221 Mpa; Model 3B: 159.488 Mpa; Model 3C: 147.231 Mpa. Statistical analysis of the compiled data was carried out. It was seen that there was a significant difference in stress values from the intact tooth Model 1 and cavity Model 2 (*p* < 0.05) with means values of 53.1 and 139.22, respectively. The means of all subgroups were comparable but there was a statistically significant difference between Model 3, i.e., 3A (67.74), Model 3B (60.47), Model 3C (53.70), and Model 2. Model 1 and Model 3C had comparable mean values.

**Conclusion:**

Rehabilitation of deep mesio-occlusal-distal cavities of molars with intact buccal and lingual walls with the aid of a horizontal post of any diameter has a similar stress distribution to an intact tooth. However, the biomechanical performance of a 2 mm horizontal post was exacting of the natural tooth. Horizontal posts can be included in expanding our restorative option for rehabilitating grossly mutilated teeth.

## 1. Introduction

Mandibular first molars are the most commonly affected teeth with dental caries due to their morphology, time of eruption, and position of the tooth in the arch. Rehabilitation of grossly mutilated vital teeth has always been a challenge [1]. Sound intact teeth rarely fracture under normal masticatory forces. All studies have emphasized enough the importance of maintaining and preserving normal structural tooth integrity. It follows as a corollary that the more extensive the preparation the weaker the tooth [2].

One of the greatest challenges in restoring deep carious lesions with mesial–distal extension is that it has decreased fracture resistance than a normal tooth [3]. Teeth with extensive cavity preparations have demonstrated greater cuspal deflection than those cavities with minimal preparations [4]. Increased concentration of stress will lead to a greater magnitude of cuspal deflections and will potentially lead to frequent loss of restoration or shearing away of tooth structure [5]. The reduced fracture resistance has been debated and discussed and inferred due to bilateral loss of marginal ridge [6].

From the biomechanical point of view, different restorative options have been investigated. Class II mesio-occlusal-distal (MOD) cavities significantly weaken the teeth, and the restorations placed in the prepared cavity should have the ability to resist the masticatory forces. A lot of research has been undertaken to determine the best replacement restorative option for vital grossly mutilated teeth [1–6]. For a long time, over a century, despite the waging discontent among the antiamalgamist, the silver amalgam ruled the roost. It was after the Minamata convention treaty was signed and honored in principle that silver amalgam was phased out. Reams and reams of paper mourned the loss of the Dark Knight and though the ideal enamel and dentin replacement material does not exist, different permutations and combinations are being explored and integrated into our day-to-day restorative practice. The predicament is that each material has different physicochemical chemical properties and may not match the vital hard tissues, further both enamel and dentin have different rheological properties.

Kuijs et al. [7] have advocated cusp replacement of the restoration and concluded that ceramic, indirect resin composite, and direct resin composite provide variable fatigue resistance. Posterior glass ionomer cement, resin-modified GIC, glass carbomer cement, bulk-fill composite materials, and alkasite-based materials have all been evaluated for restoring posteriors [1]. Studies by Kantardzić et al. [8] observed that composite material provides 83.3% fracture resistance to that of the sound intact tooth and suggested a palatal cusp reduction to lower stress values in dental hard tissues and restorations. Magne by three-dimensional finite element analysis (FEA) reviewed different restorative techniques and found that composite resin inlay resulted in partial recuperation of cuspal stabilization whereas adhesive liners resulted in complete cuspal stiffness recovery [9]. Ausiello et al. [10] observed that composite coupled in a bilayer restorative technique with posterior nonshrink high-strength glass ionomer cement had better resistance to the inherent residual stresses during different loading conditions and had reduced the shrinkage of the composite when compared with the bulk-fill composite.

Babaei et al. [11] evaluated the effect of the internal cavity design angle and the depth of restoration and observed that irrespective of the internal cavity design angle and depth of preparation the materials utilized for rehabilitating exhibited higher stress concentration which was proportional to the increased in modulus of elasticity of the material.

Grassi et al. [12] conducted a FEA to evaluate the effect on MOD cavities restored with indirect composite inlay and ceramic inlay after rehabilitating with or without deep marginal elevation with direct composite. It was observed that deep marginal elevation groups had no bearing on treatment outcome; however, groups restored with ceramic inlays exhibited a maximum concentration of stress in the restorative material. Resin composite inlays exhibited more stress concentration in the hard tissue tooth structure.

Hence, the way forward was to evaluate restorative techniques to reinforce the residual tooth structure and concomitantly reduce the stress concentration. One such proposed technique was to transfix horizontal post systems in the buccal and lingual walls of MOD cavities. It was purported to be an economical, conservative chair side technique with fairly good esthetic results, and obviated the need for full-crown coverage [13]. However, the effect of the transfixed horizontal post has not been exhaustively researched. Therefore, this study was envisaged to evaluate by three-dimensional FEA the effect of horizontal post systems as a reinforcing modality in mesiodistal cavities of vital teeth, especially when exposed to axial loads simulating mastication. The study also attempted to bridge the gap in existing evidence-based literature concerning rehabilitating MOD cavities in vital teeth.

Finite element (FE) stress analysis is a computer-generated technique that helps to better decipher the dental biomechanics of any designated geometry. FEA has a significant contribution to the development of new materials, types of equipment, techniques, and technologies in most industries including the field of biomedical research. This could be owing to the fact that a plethora of clinical simulations is performed without the requirement of the physical presence of the patients or the necessity to perform human tests [14]. A model is built with the physical and rheological properties of the biological tissues and biomaterials. Specifically, the modulus of elasticity and Poisson's ratio of the materials are needed for model build-up [7, 9, 10, 14, 15].

This study aims to examine the stress distribution under simulated masticatory occlusal loading conditions on a mandibular first molar tooth having a class II mesial–occlusal–distal (MOD) cavity with proximal box gingival floor within dentin restored with composite and rehabilitated with different diameters of horizontal posts by the three-dimensional FE stress analysis. The null hypothesis which was being examined was that rehabilitation of MOD cavities with horizontal posts of different diameters had no added benefit and different diameters had no bearing on distribution of stresses and stress patterns.

## 2. Materials and Methods

**Figure 1:**
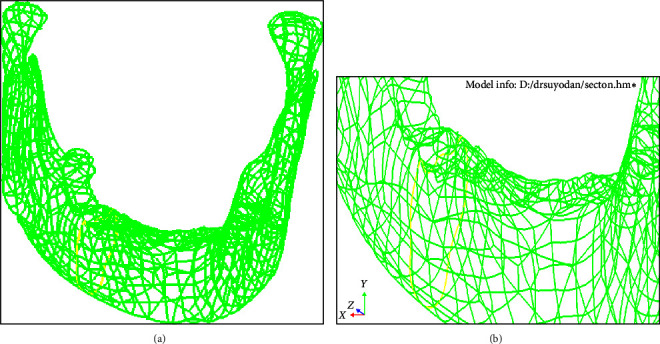
(a) Mandible with molar site; (b) section at molar region.

**Figure 2:**
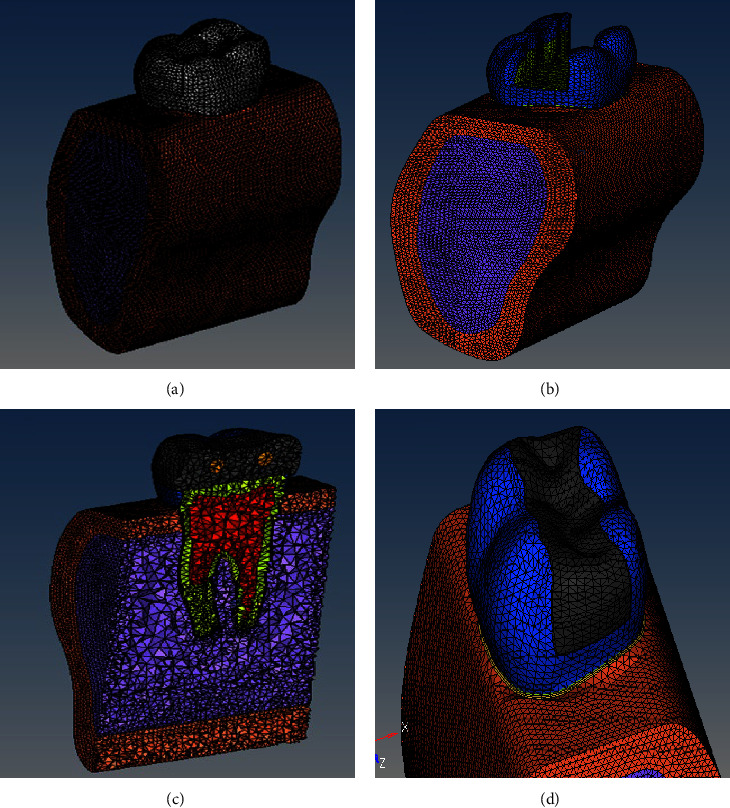
(a) Mandibular bone with molar section; (b) prepared cavity; (c) section of the restored tooth; (d) restored with composite.

A three-dimensional element model of the permanent mandibular first molar along with attachment apparatus was built and designed according to the standard anatomy as described in Wheeler's atlas [15] and based on the physical and material properties obtained from published data (Table 1) [14–19]. The tooth model which was computer-simulated based on the material properties was made up of small elements which are joined at points called nodes. Once the model was constructed, boundary conditions were made so that when stress was applied, the body under analysis was contained, hence meshing was done. The first model to be generated was the complete mandible (Figures 1(a) and 1(b)) and subsequently, the mandibular first molar was prepared and meshed (Figure 2). The mandibular molar models replicating the clinical scenarios were simulated, designed, and built (Figure 2(a)), assuming all materials to be homogenous, isotropic, and linearly elastic, based on the mechanical properties of materials, i.e., Young's modulus and Poisson ratio which was obtained from evidence-based scientific data as follows: Model 1 control: the model of an intact first mandibular molar. Model 2: the prepared cavity MOD is replicated by the subtraction Boolean method. The remaining thickness of dentin is 1 mm. Model 3: these were rehabilitated by three different diameters of two horizontal fiber posts. Model 3A: fiber post diameter 1 mm, Model 3B: 1.5 mm, and Model 3C: 2 mm. The references were defined for mandibular molar with a standardized width and depth. The depth was kept with 1 mm of the remaining thickness of dentin. The indirect pulp capping was simulated with biodentine and the thickness of pulp capping agent was 75 *μ*. The distance between the buccal and lingual wall corresponded to two-thirds of the intercuspal distance (Figure 2(b)). The distance between the two concentric holes of the horizontal fiber post is at a distance of 3 mm from the buccal fissure. The holes were 3 mm from the occlusal reference points. Different sizes of fiber posts of 1, 1.5, and 2 mm were successively used to rehabilitate the mutilated teeth. The adhesive protocol was the application of self-etch primer (Clearfil SE Bond) followed by using resin cement (Panavia 2.0) for bonding the post (Figure 2(c)). The cavity was then restored with a posterior composite (Figure 2(d)). The conversion of two-dimensional mesh to three-dimensional mesh was done using hypermesh drag options. The total elements and nodes in this study are appended in Table 2. The element type was 10-noded tetrahedral. A boundary condition was applied and then after meshing the models, boundary conditions were established and the load applied was defined on the occlusal surface on buccal and lingual distal cusps with a constant axial load value of 600 N and an angle of 45°. Thereafter three-dimensional FEA was done using ANSY software. The result of FEA is expressed as stresses, i.e., tensile, compressive, shear or a combination known as von Mises stresses. The von Mises stress is generally used in determining whether an isotropic and ductile material will yield when subjected to a complex loading condition. On the other hand, *principal stress* represents the *maximum* and minimum of normal *stresses* on a principal plane at a condition of zero *shear stress* acting on a body. While the maximum principal stress theory more accurately predicts failure, especially in brittle materials, but is often not always accurate for ductile materials [14–18]. Hence in this study, von Mises stresses were taken into consideration.

## 3. Results

**Figure 3:**
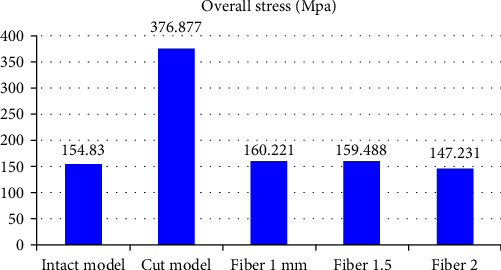
Overall stress was analyzed, and it was observed that stress distribution of fiber reinforced group was comparable to that of control but the cavity prepared group had higher stress concentration.

**Figure 4:**
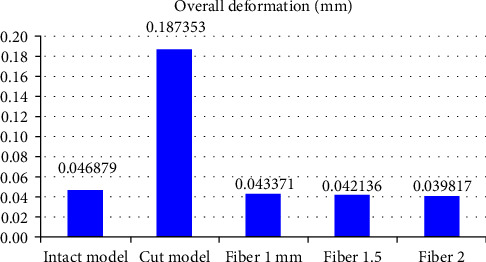
Overall deformation was analyzed, and it was observed that deformation of the fiber-reinforced group was comparable to that of the control but the cavity prepared group had higher deformation.

**Figure 5:**
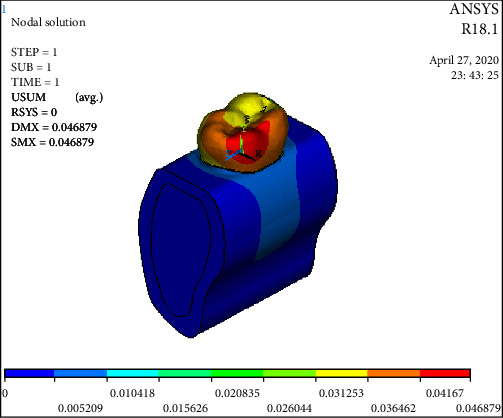
Stress analysis of intact mandibular molar shows the concentration of stress on the functional cusp.

**Figure 6:**
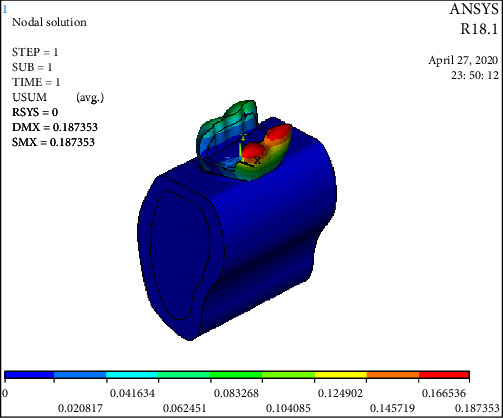
Stress analysis of mesio-occlusal-distal cavity in mandibular molar shows the concentration of stress on the functional cusp. The maximum stress is on both the functional cusps. The magnitude of stress was more as compared to the intact tooth.

**Figure 7:**
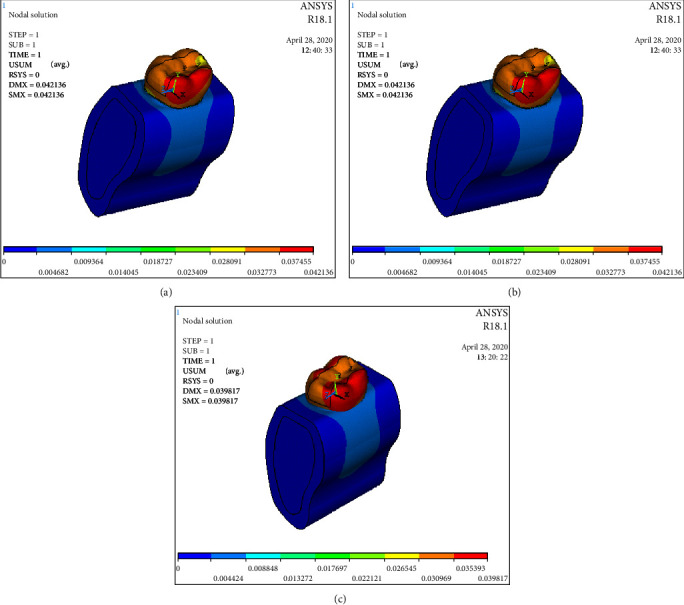
(a) The stress analysis of the 1 mm horizontal postrestored with posterior composite shows stress concentration on the whole buccal surface, including the functional cusps; (b) the stress analysis of the 1.5 mm horizontal postrestored with posterior composite shows stress concentration on the whole buccal surface, including the functional cusps. The magnitude of stresses is more than that of 1 mm; (c) the stress analysis of the 2 mm horizontal postrestored with posterior composite shows stress concentration on the whole buccal surface, including the functional cusps. The lingual cusps also show an increased concentration of stress. The magnitude of stresses is less than that of 1 or 1.5 mm horizontal posts.

The statistical analysis was conducted with parametric tests and the results were also verified by applying the nonparametric tests to the same data. The overall Von Mises stress recorded were as follows: Model 1: 154.83 Mpa; Model 2: 376.877 Mpa; Model 3A: 160.221 Mpa; Model 3B: 159.488 Mpa; Model 3C: 147.231 Mpa (Table 3 and Figure 3). Overall deformation was analyzed, and it was observed that the deformation of fiber reinforced group was comparable to that of the control but the cavity-prepared group had the highest deformation (Table 3 and Figure 4). The stress analysis for individual models was carried out. Stress analysis of intact mandibular molar showed maximum von Mises concentration on the functional cusp (Figure 5). Stress analysis of the MOD cavity in the mandibular molar exhibited the concentration of stress on the functional cusp (Figure 6). The stress analysis of the 1 mm horizontal postrestored with posterior composite shows stress concentration on the whole buccal surface, including the functional cusps (Figure 7(a)). The stress analysis of 1.5 mm horizontal post restored with posterior composite shows stress concentration on the whole buccal surface, including the functional cusps. The magnitude of stresses is more than that of 1 mm (Figure 7(b)). The stress analysis of the 2 mm horizontal post restored with posterior composite shows stress concentration on the whole buccal surface, including the functional cusps. The lingual cusps also show an increased concentration of stress. The magnitude of stresses is less than that of 1 or 1.5 mm horizontal posts (Figure 7(c)).

**Figure 8:**
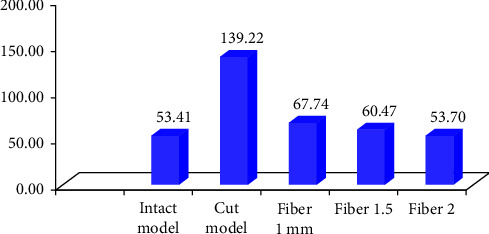
There is a nonsignificant difference if we compare mean values between models 1 and 3 and their subgroups. The mean difference is highly significant with 95% confidence and *p*-values < 0.05. Model 2 and the rest of Models 1, 3A, 3B, and 3C have similar mean values.

After the data compilation, processing, and analyzing all the stresses, the results were calculated for the models in each of the teeth occlusal and facial and lingual facets. The von Mises stress data were summarized using stress maps for the enamel tissue and dentin tissue and the restorative interfaces. It was observed that there was a significant difference in stress values from the intact tooth Model 1 and cavity Model 2 (*p* < 0.001) with means values of 53.1 and 139.22, respectively. The means of all subgroups were comparable but there was a statistically significant difference between Model 3, i.e., (3A (67.74), Model 3B (60.47), Model 3C (53.70)) with Model 2. On evaluating Model 1 and Model 3C, it was noted that they had comparable mean values. The results were also verified by applying a nonparametric test on the same data (Figure 8).

## 4. Discussion

Forces acting on teeth during mastication or parafunctional habits can lead to the generation and concentration of stresses on the sound teeth and attachment apparatus. The resultant forces become more complex, especially on the tooth restorative interfaces. Thus, before a restorative material is introduced for clinical usage, its biomechanical performance is thoroughly assessed in a simulated oral environment. Technological and software development has blurred the lines between the real-time and virtual worlds. Stress analysis of proposed technology, newer material, or an innovative technique can be evaluated and analyzed in the virtual world simulating the oral environment before it can experiment with in real time. Computer-based replication of stress analysis was first documented in the aeronautical industry and then permeated into the automobile industry and finally into medical and dental technology. The FEA is one such technique that can analyze the stress distribution of innovation before it is launched commercially or integrated into our day-to-day practice. FEA is a useful computer-based tool that can resolve differential equations by a powerful numerical technique [9, 13, 16–23].

FEA plays a pivotal role in investigations of clinical and biomechanical situations in different dental restorative scenarios [17]. The computer-generated program facilitates the computation of deformations, strains, and stresses in a distinct three-dimensional FE computed model representing a structure under static or dynamic loading on a tooth-restoration interface. Based on the physical and rheological properties of the body under analysis, the structure is computer generated. The complex body is made of multiple elements which join at nodal points called nodes. Thus, when stress has been applied, the deformation that occurs or the concentration of stress under loading conditions is analyzed and the resultant variables are calculated [16–22]. Since the dental tissues are of different constituents, of varying thickness, shape, and resiliency and more often than not nonhomogenous or symmetrical, thus they have to be simulated and replicated in three dimensions for a validated analysis [16–21]. Hence, in this study, based on evidence-based data, the finite model of an intact tooth was generated. Another model was generated of a grossly mutilated tooth which was vital with a remaining thickness of dentin of 1 mm with remaining buccal and lingual walls intact. The third model exhibited rehabilitation of the tooth with various diameters of horizontal fiber posts (Table 1, Figures 1 and 2).

Mandibular first molars have the greatest incidence of dental caries owing to their position in the arch, chronological order of eruption, and occlusal morphology. The longevity and survivability of posterior restorations are compounded by multiple factors. Forces acting on class two restorations are complex and the situation worsens when both the marginal ridges are lost, and the ensuing grossly mutilated carious teeth with MOD cavity extension are akin to a simmering cauldron of aberrant forces [1]. MOD cavities have an abysmally lesser fracture resistance than that of sound intact teeth. The decrease in fracture resistance has been attributed to the loss of marginal ridges irrespective of the quantum of tooth removal [1]. This could be the very reason that in such clinical scenarios, the recoup of fracture resistance is meager as compared to that of a natural tooth, no matter what would be the means of rehabilitation or restoration. A plethora of materials, including the phased out silver amalgam, different types of composites, ceramics, composites with ceramic inserts, restorative glass ionomers, and resin-modified glass ionomers have all been attempted as a posterior replacement material but the outcome has been far from predictable. Various permutations and combinations of different direct and indirect methods of restoration have been attempted with variable results, the search for an ideal material which has a similar modulus of elasticity to the lost material is still being researched and is ongoing. Thus, an innovative method is to be explored which can have a lintel canopy effect akin to the roof of a building. A lintel is a horizontal rod-like structure that is integral on rooftops and above windows and doors. Its primary aim is to support, reinforce, and transfer the load to the side walls. Thus, in simple terms, they are deployed for load bearing to dissipate the load and stress. In the construction of a building, the reinforced concrete mixture is intertwined and interspersed with iron bars or rods, rechristened rebar, with the sole aim of strengthening the superstructure.

Taking a cue from the construction industry, this paper has evaluated a method of rehabilitating vital teeth which has only the buccal and lingual wall remaining in situ. The horizontal posts are placed in the preparation placed at a distance of 2–3 mm from the central buccal fissure and 2–3 mm distance from the occlusal reference mark. The values may be variable depending on the availability of sound tooth structure. The horizontal placement technique was similar to the documented case reports and review of the literature [23, 24]. Thus, to improve the structural integrity, a canopy of fiber posts was used for reinforcement to deflect incident masticatory loads and prevent unrepairable catastrophic fractures.

Plotino et al. [25] observed that there was a sharp dip in fracture resistance of 44% with indirect composite and 42% when direct composite was used. Conversely, Bassir et al. [26] found contrary results, they observed an improvement in fracture resistance improvement when cuspal coverage was attempted with either direct or indirect restorative option. At times, the results replicated intact tooth stress distribution patterns. On the other hand, teeth that were rehabilitated with an onlay had a homogenous distribution of stress as compared to inlay. Further, when composites were used either indirectly or as a direct restorative material, they lead to an increase in fracture resistance as compared to the intracoronal restoration [7]. Stappert et al. [27] drew an inference that only functional cusp coverage could improve fracture resistance as compared to full coverage by ceramics. Thus, there is no consensus on the fracture resistance of restored teeth [8, 17–24]. In the present study, it was observed that there was an exponential increase in deformation and overall stress pattern in a MOD cavity when compared to a sound intact tooth. The rehabilitation by horizontal posts of different diameters was analyzed. It was observed that all three different diameters of the horizontal post could bring about total stress distribution in the same range as that of the intact sound tooth. Further, it was noted that the 2 mm diameter of the fiber post had stress values closest to that of the control tooth.

Ferri et al. [28] evaluated the fracture resistance of endodontically treated teeth rehabilitated with horizontal posts and studied the different effective positions of fiber posts. It was observed that there was no statistical difference between fiber posts placed in the middle third or 2 mm below the middle third. However, the middle third placement exhibited a lower incidence of pulpal floor fracture than when restored with composites alone. In this study, since the tooth was vital the horizontal post was kept at a constant position, i.e., 2 mm below the occlusal reference and the variation in diameters was only assessed it was observed that 2 mm of the horizontal post gave results comparable to that of the intact tooth. Further, all diameters performed better in terms of stress distribution as compared to the MOD cavities restored with composite.

Borges et al. [29] evaluated the concentration and distribution of stress endodontically treated teeth with transfixed glass fiber posts. The different simulated conditions which were evaluated were composite resin as control, vertical fiber posts for rehabilitation, transfixed glass fiber posts used alone, and a combination of vertical glass fiber posts and transfixed horizontal posts. The loading condition included 300 N applied on the occlusal surface. The authors inferred rehabilitation with horizontal glass fiber posts did not reduce any stress and in fact the stress concentration was similar to conditions in which no posts were given in endodontically treated teeth. Further, the author did not elucidate the diameter of the transfixed post. In this study, too transfixed fiber post was assessed for stress distribution during mastication but the effect of different diameters of fiber post was also evaluated. The main difference in both studies was that in Borges et al.'s [29] study, the stress generation pattern was assessed in endodontically treated teeth, but in our study, the study groups were vital teeth with mesiodistal cavities. As the teeth were vital, the pattern of biomechanical stress generation, distribution, and cuspal deflection is incomparable. Further, in this study, it was observed that the stress generated and distribution with an increasing diameter was similar to that of an intact tooth.

Thus, a horizontal post can be a viable option compared to complete restoration with composites. However, since it is a computerized study, FEA has some inherent limitations. FEA can handle complex geometries but for a vital tissue, a closed-form mathematical solution is difficult to obtain. The result is at best an approximation as the FEA is not performed on live tissue. The output which is obtained is only as good as the input rendered by the investigator. It is not immune to or free from the GIGO phenomenon. GIGO is an acronym for “Garbage In → Garbage Out” which means and signifies that a bad input will result in a bad output [30]. In this study, the limitations of FEA have been taken into consideration. The different types of fiber post diameters have been selected and analyzed, different clinical scenarios like margin elevation, remaining dentin thickness also need to be analyzed and will be taken up in future studies. Nevertheless, it can be at best simulate clinical scenarios but cannot replicate clinical conditions in toto. Hence, when the results are being scrutinized, this shortcoming should be taken into account before clinical translation.

## 5. Conclusion

Rehabilitation of deep MOD cavities of vital molars with intact buccal and lingual wall with the aid of horizontal post of any diameter has a similar stress distribution of an intact tooth. However, the biomechanical performance of a 2 mm horizontal post was exacting of the natural tooth. Horizontal posts can, thus, be included in expanding our restorative option for rehabilitating grossly mutilated vital teeth.

## Figures and Tables

**Table 1:** Material properties [13–19].

Description	Material properties	
	Modulous of elasticity—GPA	Poisson's ratio
Enamel	41	0.3
Dentin	18.6	0.31
Pulp	0.23e−7	0.3
Periodontal ligament	68.0e−3	0.45
Cortical bone	13.7	0.3
Cancellous bone	1.37	0.3
Gingiva	19.6e−3	0.3
Fiber post	*X* = 37, *Y* = 9.5, *Z* = 9.5	0.26
Posterior composite—filtek bulkfill	1460.22 ± 97.20 MPA	0.39
Resin cement—Panavia 2.0	18.6	0.28
Clearfil SE bond	0.56	0.25
Biodentine	22.0	0.3

**Table 2:** Element and nodes of different models.

	Elements	Nodes
Model1 (Intact)	383,333	553,392
Model 2 (cut)	374,970	542,601
Model 3a (1 mm fiber)	378,982	548,646
Model 3b (1.5 mm fiber)	383,201	552,652
Model 3c (2 mm fiber)	387,061	558,499

**Table 3:** Stress and deformation comparison for all models.

Description	Intact model	Cut model	Fiber 1 mm	Fiber 1.5	Fiber 2
Overall deformation	0.046879	0.187353	0.043371	0.042136	0.039817
Overall stress (Mpa)	154.83	376.877	160.221	159.488	147.231
Cortical stress (Mpa)	17.06	21.9927	16.5746	16.5201	16.4139
Cancellous stress (Mpa)	9.66112	11.1289	9.24099	9.16383	9.05681
Dentine stress (Mpa)	80.7399	313.074	77.5838	77.0741	76.4912
Enamel stress (Mpa)	154.83	376.877	160.221	159.488	147.231
Biodentine	–	–	78.713	78.541	78.011
Peristress (Mpa)	10.083	13.6106	9.43361	9.24797	9.02103
Pulp stress (Mpa)	0.000000542	0.00000056	0.000000537	0.000000535	0.000000534
Fiber stress	–	–	107.597	85.5293	78.426
Composite			136.461	88.1937	53.1284

## Data Availability

All data generated or analyzed during this study are original and are included in this published article. All data will be shared by the author on request by contacting the corresponding author.

## Additional Points

*Clinical Significance*. Grossly mutilated teeth rehabilitation is a challenge. Full coverage restorative options further reduce the tooth structure. Rehabilitation with horizontal post systems will lead to better stress distribution and better accessibility in case endodontic intervention is needed.
